# Interactions of Cyclic Peptides Ribifolin and Gramicidin S with Montmorillonite Surface by Molecular Modeling

**DOI:** 10.1021/acsomega.5c13251

**Published:** 2026-03-19

**Authors:** Lucas H. N. Sousa, Claro Ignacio Sainz-Díaz, César Viseras, Renata M. Araújo

**Affiliations:** † Instituto de Química, 28123 Universidade Federal do Rio Grande do Norte, Natal 59078-970, Brazil; ‡ Instituto Andaluz de Ciencias de la Tierra, 16379 Consejo Superior de Investigaciones Científicas, IACT-CSIC, Av. De las Palmeras, 4, 18100-Armilla, Granada 18100, Spain; § Departamento de Tecnología Farmacéutica. Facultad de Farmacia, 16741 Universidad de Granada, Granada 18071, Spain

## Abstract

Cyclic peptides are characterized by remarkable structural stability and considerable therapeutic potential, but their clinical application is often limited by enzymatic degradation, especially in oral administration. Recent approaches, including the design of small cyclic peptides and clay-based delivery systems, aim to improve protection, bioavailability, and controlled release. Among these carriers, layered clay minerals, such as montmorillonite (MONT), are particularly attractive. However, the development of peptide–clay formulations remains largely empirical, since the molecular mechanisms governing adsorption, intercalation, stabilization, and release under hydrated and confined conditions are difficult to probe experimentally. Atomistic simulations using the INTERFACE force field are applied as a preformulation screening strategy to anticipate the behavior of the cyclic peptides ribifolin and gramicidin S at MONT interfaces prior to experimental implementation. Neutral and protonated states are examined, including pH-dependent cation-exchange scenarios relevant to the formulation conditions. The methodology is validated against experimental crystal structures, reproducing lattice parameters with deviations below 6% and demonstrating computational reliability. Protonation enhances peptide stabilization in aqueous media. Energetic analyses show that adsorption on external MONT surfaces is favored over interlayer intercalation, which is predominantly endothermic, whereas desorption into water is exothermic and indicates efficient release under physiological conditions. Although the calculated energies are not full thermodynamic values, the trends reveal how peptide–clay affinities can direct formulation experiments and minimize empirical testing. By integrating molecular modeling with pharmaceutical design, this study supports montmorillonite as a rational platform for the future experimental development of cyclic peptide delivery systems.

## Graphical abstract

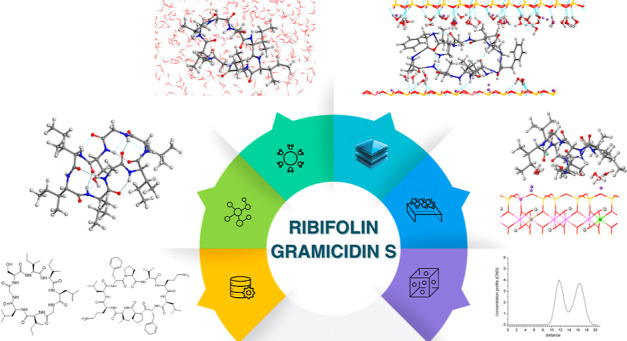

## 1 Introduction

Cyclic peptides are defined by a covalently closed peptide backbone that restricts conformational freedom and eliminates terminal functional groups, leading to enhanced structural stability and a well-defined three-dimensional distribution of functional moieties. They are produced by terrestrial and marine organisms or obtained synthetically through approaches such as solid-phase peptide synthesis (SPPS).^1–3^ In particular, nonribosomal biosynthetic pathways generate chemically diverse architectures with rigid conformations and increased resistance to chemical and enzymatic degradation.^4–6^

As a result of these structural and biosynthetic features, cyclic peptides have emerged as attractive therapeutic scaffolds. They combine structural stability with controlled exposure of polar and hydrophobic residues, thereby influencing solubility, membrane interaction, and permeability. Consequently, cyclic peptides are actively explored for drug development, where high selectivity, potency, and tunable bioavailability may offer advantages over linear peptides and small-molecule drugs.^3,6,7^ Reported applications include metal sequestration, detoxification, and a wide range of biological activities, such as antimicrobial, immunosuppressive, cytotoxic, anti-inflammatory, and antimalarial effects.^8–11^

Oral administration of cyclic peptide drugs is attractive as a noninvasive, low-cost route, and, in some cases, cyclic architectures show enhanced membrane permeability relative to linear peptides by adapting conformation and hydrophobicity to the environment.^12–14^ Nevertheless, gastric and intestinal enzymes, together with variable pH conditions, can induce peptide cleavage or conformational destabilization, reduce bioactivity, and often require parenteral administration.^7^ To address these limitations, strategies include small cyclic peptide design for improved permeability and protective delivery systems for controlled release.^15^

In this context, natural clay minerals are appealing carriers due to their low toxicity, biocompatibility, high surface area, and ion-exchange capacity, which allow stable yet tunable interactions with drug molecules.^16,17^ Drug incorporation by adsorption on external surfaces or intercalation within the layered clay structure can reduce direct exposure to the gastrointestinal environment, stabilize bioactive conformations, and modulate the release kinetics. In addition, exchangeable cations and interlayer water control peptide affinity and mobility, enabling gradual transfer of the active compound into the surrounding medium.^18–20^

Phyllosilicate minerals exhibit distinctive physicochemical properties associated with their layered structure, permanent charge, and formation of fine particles, often with dimensions smaller than 2 μm. These features strongly influence how hydrated aluminosilicate layers interact with peptides through electrostatic forces, hydrogen bonding, and confinement effects. However, the small crystal size of clays combined with peptide flexibility and solvent organization makes it difficult to accurately identify and characterize host–guest interactions, which is crucial for the development of controlled drug delivery systems.^21,22^

Molecular modeling enables an atomic-level investigation of these processes, providing detailed descriptions of organic–inorganic interfaces, preferred binding sites, orientations, and interaction energies often inaccessible to experiments alone. Although the calculated energies are not full thermodynamic quantities, their trends reveal how peptide–clay affinities can guide formulation strategies and minimize empirical testing. Thus, integrating in silico tools optimizes experimental efforts by reducing resource consumption and enabling preselection of relevant adsorption environments and chemical conditions.^23–25^ Therefore, crystal-derived conformations serve as experimentally grounded structural references for confined peptide states, supporting the identification of preferential adsorption sites, stable orientations, and key interaction motifs at the molecule–clay interface. Molecular dynamics and force-field approaches further improve understanding of organic adsorption on hydrated clay mineral surfaces and interlayers.^26–32^

In this work, the interaction of two cyclic peptides with montmorillonite (MONT) as a model clay carrier was investigated using molecular modeling approaches relevant to controlled drug delivery systems. The molecular structures of the peptides are analyzed in both neutral and protonated states, and their behavior under confinement within the MONT interlayer is examined to capture pH-dependent and ion-exchange effects typical of formulation environments.^33,34^

Ribifolin is a hydrophobic cyclic orbitide (Figure 1a) obtained from *Jatropha ribifolia* (Pohl) Baill. and synthesized by SPPS. Its structure has been characterized by mass spectrometry (MS) and nuclear magnetic resonance (NMR) spectroscopy and resolved three-dimensionally by racemic crystallography, revealing moderate antimalarial activity against *Plasmodium falciparum*.^10,35,36^ Gramicidin S is a cyclic, 2-fold symmetric amphiphilic decapeptide (Figure 1b) that adopts a stable β-turn and antiparallel β-sheet arrangement stabilized by intramolecular hydrogen bonds. It is biosynthesized by bacteria such as *Bacillus brevis*, *Aneurinibacillusmigulanus*, and *Aneurinibacillus aneurinilyticus*, and its crystal structure has been determined by X-ray diffraction (XRD), MS and NMR. Although its hemolytic toxicity restricts systemic use, gramicidin S is widely employed as a topical antibiotic and serves as a relevant model for studying peptide stability, adsorption, and release at inorganic interfaces.^37–48^

**Figure 1:**
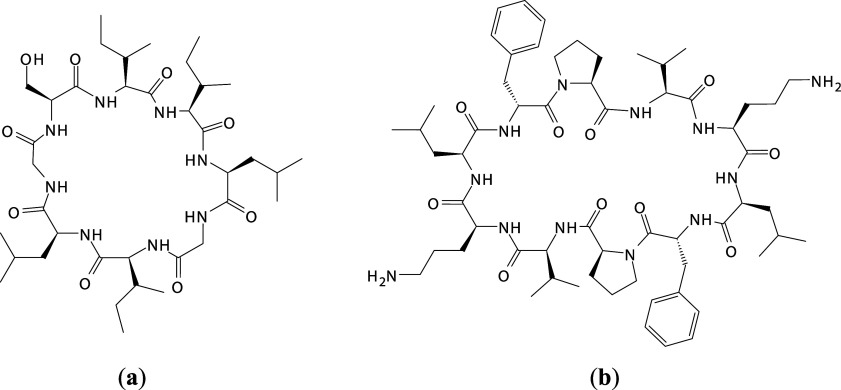
Molecular structure of the cyclic peptides: (a) ribifolin; (b) gramicidin S.

This study investigates how peptide structures interact with the surfaces and interlayers of MONT under formulation-relevant conditions when structurally different cyclic peptides are present. The computational methodology was validated by comparison with experimental crystal structures of both cyclopeptides by applying the INTERFACE force field, with lattice parameter deviations below 6%. The results indicate that ribifolin and gramicidin S can be adsorbed onto MONT surfaces and exhibit an energetically favorable transfer to the aqueous phase, which corroborates their potential for administration from a clay carrier.

## 2 Materials and Methods

### 2.1 Crystal Structure Models

The experimental crystal structures of the cyclic peptides were retrieved from the Cambridge Crystallographic Data Centre (CCDC) database. The corresponding crystallographic information files (CIFs) were identified by deposition numbers 1861364, corresponding to the l-isomer of ribifolin [cyclo­(glycyl-seryl-isoleucyl-isoleucyl-leucyl-glycyl-isoleucyl-leucyl) monohydrate],^10^ and 1,870,209, to the gramicidin S hydrochloride monohydrate [cyclo­(leucyl-phenylalanyl-prolyl-valyl-5-azaniumylnorvalyl-leucyl-phenylalanyl-prolyl-d-valyl-5-azaniumylnorvalyl) dichloride monohydrate].^43^ System validation was performed by comparing the calculated lattice cell parameters with the experimentally determined values reported in the original crystallographic studies.

For the calculations, a periodic crystal structure model of montmorillonite (MONT) was employed,^49–51^ an essential constituent of Veegum, a pharmaceutical grade clay mineral widely used in therapeutic formulations.^52,53^ In this dioctahedral phyllosilicate, the octahedral sheet is composed predominantly of Al^3+^cations with minor substitution by Mg^2+^, whereas the tetrahedral sheet consists mainly by Si^4+^ cations with a small substitution by Al^3+^. These isomorphic cation substitutions cannot be determined with high precision by XRD. Therefore, they were modeled with a maximum spatial dispersion along the sheets.^54^ Empirical interatomic potential calculations were used to optimize manually positioned hydrogen atoms.^27^ The chemical composition of a MONT unit cell of this clay mineral is Na­(Si_7.83_Al_0.17_)­(Al_3.17_Mg_0.83_)­O_20_(OH)_4_. Supercells were constructed for peptide adsorption simulations, balancing computational cost and minimizing additional interactions with vicinal cells in three-dimensional periodic systems. Accordingly, 3 × 2 × 1 (ribifolin) and 6 × 4 × 1 (gramicidin S) supercells, comprising 1302 atoms, were created and used in the adsorption studies. The interlayer Na^+^ cations were solvated with two water molecules each. To model an external surface, the lattice parameter c was set to 40 Å, ensuring sufficient separation to prevent surface–surface interactions.

### 2.2 Calculation Methodology

Computational atomistic modeling was carried out using the Materials Studio package, employing approaches based on empirical force field (FF) methods.^55^ The INTERFACE FF was selected due to its demonstrated reliability in describing systems containing clay minerals, organic compounds, and hybrid materials.^27,33,56–58^ For comparison purposes, calculations were also performed using the COMPASS III FF.^59^ Geometry optimizations were conducted with the Forcite code within the Materials Studio package under three-dimensional periodic boundary conditions. Electrostatic interactions were evaluated by Coulombic potentials defined in the FF, while van der Waals interactions were described by the Lennard-Jones potential. Both nonbonded interactions were treated using the Ewald summation method. Atomic positions were optimized without geometric restraints and constraints, and net atomic charges were assigned using the charge equilibration (QEq) method.^60^ The simulations considered the peptide in both neutral and protonated states; in the latter, a chloride counterion was placed near the ammonium group to ensure overall charge neutrality. Molecular dynamics simulation was performed in the canonical (NVT) ensemble at 298 K using a Nosé thermostat with a time step of 1 fs and a total simulation time of 50 ps. Configurations were sampled every 100 steps (500 samples).

To estimate peptide affinity in aqueous media, periodic simulation boxes containing 400 water molecules were constructed using a simulated annealing Monte Carlo approach, within the Material Studio package, at 298 K, where the molecules are introduced randomly with a maximum number of 10,000 attempts made to load all molecules into the cell. The 3-D box dimensions are adjusted to each peptide, the number of water molecules, and a target density of 1 g cm^–3^. Independent optimizations were performed for systems containing only water, solvated NaCl, or the peptide. For neutral species, adsorption energies were obtained from the energy difference between the optimized peptide–MONT complex and the isolated optimized components. For protonated species, the energetic contribution of chloride counterions required for charge neutrality was explicitly included. Desorption energies from MONT to the aqueous medium were estimated by comparing peptide solvation energies in water with their corresponding adsorption energies on MONT surfaces.

The resulting adsorption, solvation, and desorption energies represent effective interaction energies derived from optimized structures and molecular dynamics sampling. These values are not intended to provide a complete thermodynamic decomposition of the adsorption or intercalation processes but rather to serve as comparative descriptors of peptide–clay affinity under different chemical environments.

## 3 Results and Discussion

### 3.1 Structures of Cyclic Peptides

The force fields were validated comparing our results with experimental crystallographic structures provided from the CCDC, including high-resolution data for ribifolin (l isomer) and a refined crystal structure of gramicidin S.^10,43^ Full optimization of atomic positions and unit cell parameters yielded values in close agreement with experiments with relative deviations below 6% for all INTERFACE-analyzed parameters (Table 1). These results indicate that the selected force field accurately reproduces, within the expected precision, intramolecular geometries, cyclic topology, and average crystal packing, supporting its application to cyclopeptide modeling. Notably, the reliability of molecular simulations in reproducing experimental observables and generating predictive insights critically depends on appropriate force field selection and validation.^61–64^ In contrast, COMPASS exhibited larger deviations, indicating an inferior performance.

**Table 1:** Comparison of Experimental and Calculated Crystal Lattice Cell Parameters of Ribifolin l-isomer and Gramicidin S Hydrochloride Monohydrate (Distances in Å and Angles in Degrees)

lattice parameters		*a*	*b*	*c*	α	β	γ
ribifolin	experimental^10^	19.142	23.632	23.851	90.0	90.0	90.0
	COMPASS^a^	18.454(3.6)	24.333(3.0)	21.235 (11)	90.0	90.0	90.0
	FF INTERFACE^a^	19.587 (2.3)	22.840(3.3)	22.526(5.6)	90.0	90.0	90.0
gramicidin	experimental^43^	34.988	23.381	18.866	90.0	90.0	90.0
	FF INTERFACE^a^	34.296 (2.0)	22.957(1.8)	18.218(3.4)	89.1	89.7	90.2

a The relative deviations (%) are in brackets.

Validation against crystal structures is a stringent parametrization test, as it requires a balanced description of both intramolecular and noncovalent interactions in the solid state.^65,66^ The correct reproduction of bond lengths and angles, lattice parameters, ring conformation, and intermolecular interactions demonstrates that the force field captures the essential electrostatic, van der Waals, and bonded contributions governing system stability.^67,68^

Cyclopeptides were initially modeled in their neutral form, as their cyclic chain precludes a classical zwitterionic state despite possible localized charges along the backbone or side chains. For ribifolin, the experimental structure corresponds to a neutral monohydrate crystal comprising eight peptide molecules and four water molecules per unit cell. The optimized isolated structure retained the cyclic topology, stabilized by a defined network of intramolecular backbone hydrogen bonds and hydrophobic interactions among nonpolar residues (Figure 2a). To evaluate the protonated state, ribifolin was also modeled as a monochloride salt. The structural optimization preserved the ring conformation and native intramolecular interactions, while introducing additional stabilizing peptide–anion contacts (Figure 2b), indicating that counterion association enhances electrostatic stabilization without compromising ring integrity.^69^

**Figure 2:**
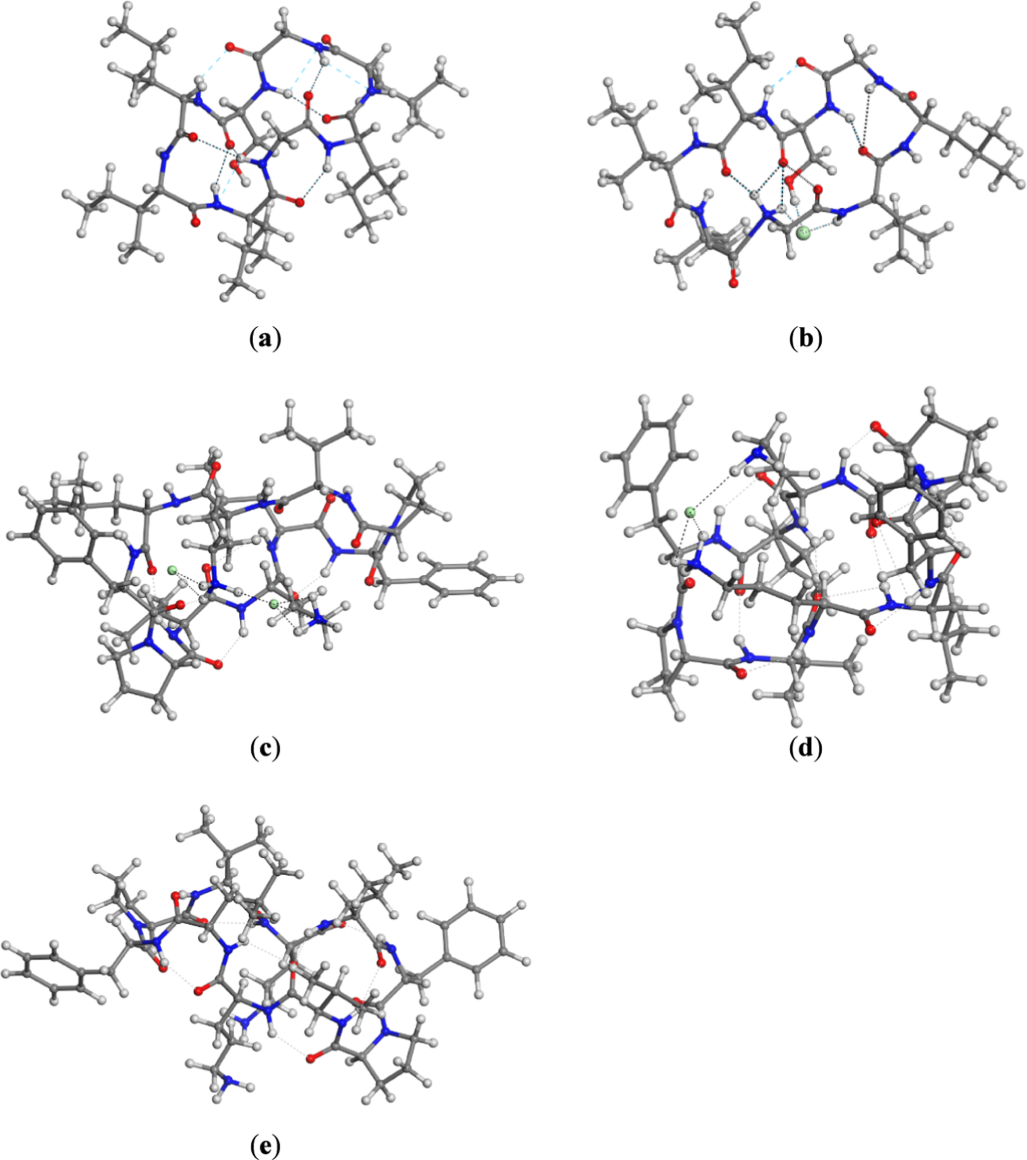
Optimized molecular structures (dashed lines indicate interactions with chloride ion or selected nonbonded intramolecular interactions. Atom colors: H-white; C-gray; O-red; N-blue; and Cl-green): (a) ribifolin in neutral state; (b) ribifolin monohydrochloride; (c) gramicidin S dihydrochloride; (d) gramicidin S monohydrochloride; and (e) gramicidin S in neutral form.

In the crystalline phase, gramicidin S displays higher ionic complexity, occurring in a doubly protonated form neutralized by two chloride counterions and lattice-associated water molecules. Based on the experimental structure, the decapeptide was isolated and optimized in dihydrochloride, monohydrochloride, and neutral states within three-dimensional periodic boxes (Figure 2c–e). Under all conditions, the stabilization features are consistent with those identified for ribifolin, suggesting a common structural behavior among orbitides.

Although crystal structures describe specific thermodynamic minima influenced by packing effects, solvents, and counterions, they offer a robust and experimentally grounded structural reference rather than a direct depiction of solution-dominant conformations.^70–72^ In studies involving clay-based delivery systems, this solid-state framework is not merely an artificial constraint but reflects the functional environment governing molecular confinement, stabilization, and release. Crystal-derived conformations therefore provide a valuable starting point for computational modeling, enabling the identification of preferential adsorption sites, stable orientations, and key interaction motifs at the molecule–clay interface.^73–76^ When integrated energy calculations and molecular dynamics simulations within the MONT interlayer, this approach yields a physically consistent and statistically convergent description of cyclopeptide behavior, supporting hypotheses on stability, bioactivity, and controlled release that are difficult to access experimentally due to molecular flexibility and nanoscale heterogeneity.^77,78^

### 3.2 Cyclic Peptide Interactions

#### 3.2.1 Solvation in Aqueous Media

Solvation of the neutral and protonated peptide species was simulated within cubic cells of approximately 24 × 24 × 24 Å^3^, each containing 400 water molecules. The peptide was positioned in the center of the simulation box. Three-dimensional periodic boundary conditions were applied with the lattice parameters maintained fixed, ensuring that the unit cell dimensions were sufficiently large to prevent significant interactions between peptides in neighboring periodic cells (Figure 3). After constructing the amorphous mixtures of the peptide with water molecules, the most stable frame was selected and optimized with the interface FF.

**Figure 3:**
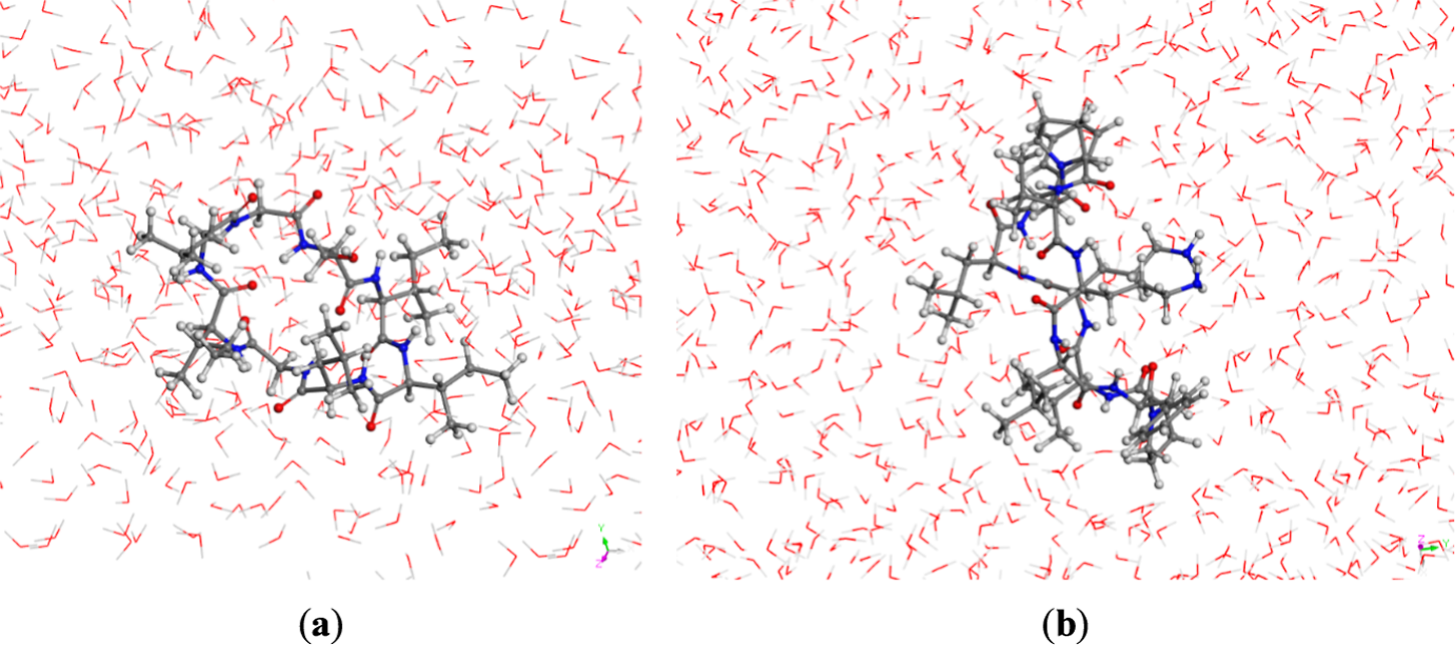
Optimization of water-solvated peptides: (a) ribifolin; (b) gramicidin S.

The solvation energy (*E*_solv_), defined in eq 1 as the difference between the optimized solvated system (*E*_w‑pep_) and the sum of the optimized isolated peptide (*E*_pep_) and water box (*E*_w_), was exothermic in all cases with more negative values observed for the protonated (hydrochloride) forms. These results indicate an increased stabilization of charged species in aqueous media, consistent with stronger peptide–solvent electrostatic interactions (ribifolin: −121.59 kcal/mol; ribifolin monohydrochloride: −167.51 kcal/mol; gramicidin S: −119.28 kcal/mol; gramicidin S monohydrochloride: −149.51 kcal/mol; gramicidin S dihydrochloride: −197.59 kcal/mol)

1 $$E_{solv}=E_{w‐pep}-\left(E_{w}+E_{pep}\right)$$

#### 3.2.2 Intercalation into MONT Interlayer

The adsorption capacity of the MONT interlayer surface toward the peptides was evaluated by using a model comprising 12 water molecules coordinating the interlayer sodium cations (two water molecules per Na^+^) and an interlayer spacing of 20 Å along the *c*-axis. The peptides were positioned at the center of the interlayer region, with their cyclic backbones oriented parallel to the (001) plane of the mineral surface. Distinct supercells were constructed for each peptide: 3 × 2 × 1 for ribifolin and 6 × 4 × 1 for gramicidin S. These differences arise from the distinct cross-sectional dimensions of the optimized peptide structures, reflecting their octapeptide and decapeptide nature, respectively. In all cases, the models were designed to be as compact as possible while avoiding spurious interactions between periodic images in neighboring cells.

In all cases, intercalation of the cyclic peptides resulted in an increase in the *d*(001) interlayer spacing. This expansion was slightly more pronounced for the neutral peptide forms (Table 2). A progressive decrease in the interlayer spacing was observed with an increase in peptide charge, which can be attributed to stronger electrostatic host–guest interactions. For the intercalation of neutral and hydrochloride forms of ribifolin, molecular dynamics (MD) simulations were performed in order to explore alternative and potentially more stable configurations. The first 10 ps was used for equilibration, followed by a 40 ps production run. The most stable configuration was selected from the MD trajectory and subsequently optimized. The peptide conformation and its orientation within the interlayer region were found to be similar in simulations performed with and without MD.

**Table 2:** Lattice Cell Parameters and Interlayer *d*(001) Spacing of Optimized MONT–Peptide Adsorption Complexes (Distances in Å and Angles in Degrees)

lattice parameters^a^		*a*	*b*	*c*	α	β	γ	*d*(001)
MONT		15.48	17.88	12.30	96.5	107.9	90.1	11.70
ribifolin	neutral	15.48	17.88	21.04	96.9	111.5	90.1	19.58
	monochloride	15.49	17.88	18.09	98.3	110.8	90.1	16.91
gramicidin S	neutral	30.98	35.79	22.39	105.7	109.1	90.1	21.16
	monochloride	30.98	35.78	22.17	103.4	113.8	90.1	20.28
	dichloride	30.97	35.78	20.67	94.5	110.0	90.1	19.42

a Optimized sample after molecular dynamic simulation.

The optimized structures of the peptides intercalated within the confined interlayer space of MONT (Figure 4a,b), as well as those involving cation-exchange configurations (Figure 4c–e), did not exhibit significant deviations from the initial unit–cell parameters. Only minor contractions, expansions, or angular variations were observed. In all cases, the cyclic peptide framework was preserved and adopted a compact, globular conformation within the interlayer region, displacing the Na^+^ cations toward the mineral surface. These cations were coordinated to the basal oxygen atoms on the mineral surface. The globular form was maintained with the help of intramolecular hydrogen bonds between the NH H atoms and the carbonyl O atoms of the amino acids. In ribofilin, the OH H atoms formed hydrogen bonds with the basal tetrahedral O atoms of mineral surface (Figure 4a), whereas the NH H atoms formed hydrogen bonds with the mineral surface O atoms in gramicidin (Figure 4b), especially the ammonium groups of the gramicidin dichloride (Figure 4e). The water molecules formed hydrogen bonds with the basal O atoms of the MONT interlayer surface, and some of them coordinated Na^+^ cations.

**Figure 4:**
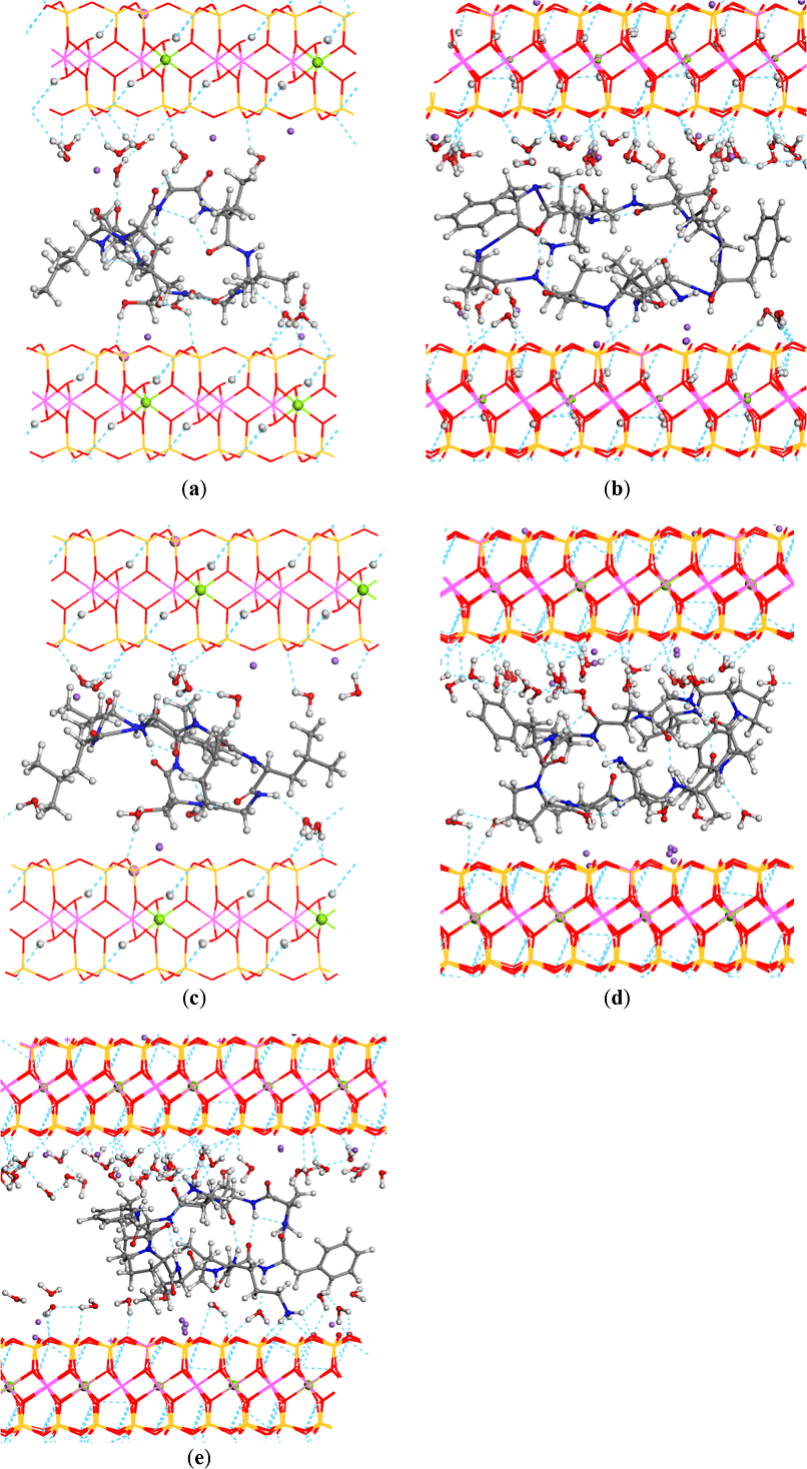
Optimized structures of cyclic peptides intercalated into the interlayer space of MONT (atom colors: H-white; C-gray; O-red; N-blue; Cl-mint green; Na-purple; Al-harlequin green): (a) ribifolin; (b) gramicidin S; (c) cation exchange of ribifolin monochloride; (d) cation exchange of gramicidin S monochloride; and (e) cation exchange of gramicidin S dichloride. The hydrogen bonds are highlighted by dash lines. The cation substitutions of Mg^2+^ in the octahedral and Al^3+^ in the tetrahedral sheets of MONT are highlighted as balls.

The cyclic structures of ribifolin (Movie M1 in Supporting Information) and gramicidin (Movie M2 in Supporting Information) were maintained during the MD simulations showing highly flexible structures, where the cyclic disposition was highly deformed in each step of the simulations without breaking the cycle. The diffusion coefficients of the atoms intercalated into the confined interlayer space of MONT were calculated from mean square displacement (MSD) analyzed from the MD trajectories collected during the production phase (400 samples). To characterize the mobility of the cyclic peptide, we can consider the main atoms of peptide (CNO), the C, N, and O atoms of peptides (Figure S1). We also considered the Na^+^ cations and water O atoms for comparison. For ribifolin intercalated in MONT, the diffusion coefficient of the peptide (C + N + O atoms) was 0.062 Å^2^/ps, being 0.074 Å^2^/ps for C atoms, 0.027 Å^2^/ps for N atoms, and 0.034 Å^2^/ps for the peptide O atoms. The reduced mobility of these N and O atoms can be attributed to the intramolecular interactions that preserve the cyclic structure of the peptide. Nevertheless, these mobilities were similar to that of water O atoms (0.042 Å^2^/ps) and much higher than that of Na^+^ cations (0.0006 Å^2^/ps). Similar values were found with hydrochloride ribifolin, 0.067 Å^2^/ps for C + N + O, and 0.068 Å^2^/ps for C atoms. In gramicidin, the mobility was lower due to steric interactions with the bigger molecular size with respect to ribifolin, being 0.009 (CNO), 0.0113 (C), and 0.0015 (N) Å^2^/ps.

During the MD simulations, we analyzed the concentration profile of the intercalated peptide atoms along the 001 direction of the crystal structure of MONT. The ribifolin remained centered within the MONT interlayer region, with the atoms located at an average minimal distance of approximately 3 Å from the mineral surface and distributed in a disordered manner along the interlayer space. Two maxima were observed in a broad concentration profile for the main peptide atoms (C + N + O) (Figure S2a) and C atoms (Figure S2b), confirming the disordered distribution. The middle valley observed in this profile indicated the loop of the cyclic structure of ribifolin. This behavior was also observed in gramicidin with a bipolar distribution that is more remarked due to the loop of the cyclic structure that in gramicidin is more ordered than in ribifolin (Figure S3). The concentration profile for the peptide N atoms also indicated a centered disposition of ribifolin showing 3 maxima (Figure S2c), whereas the profile of the peptide O atoms showed an anisotropic distribution into the interlayer space of MONT (Figure 2d). This behavior is observed for both peptides and for both the neutral and cationic states.

#### 3.2.3 Adsorption onto the External Mineral Surface

An alternative adsorption site was the outer surface of MONT, which represents the interstitial regions and nanopore surfaces of the clay mineral. To model this scenario, the external surface was simulated by introducing a 20 Å vacuum layer normal to the (001) plane of MONT (Figure 5). In general, the cyclic structure was maintained by intramolecular hydrogen bonds, being more ordered in gramicidin than in ribifolin. No strong nonbonding interactions were observed between peptide and mineral surface, being mainly electrostatic and van der Waals interactions. However, the gramicidin dichloride molecule showed a strong hydrogen bonds between one ammonium group and the basal tetrahedral O atoms of the mineral surface (Figure 5e).

**Figure 5:**
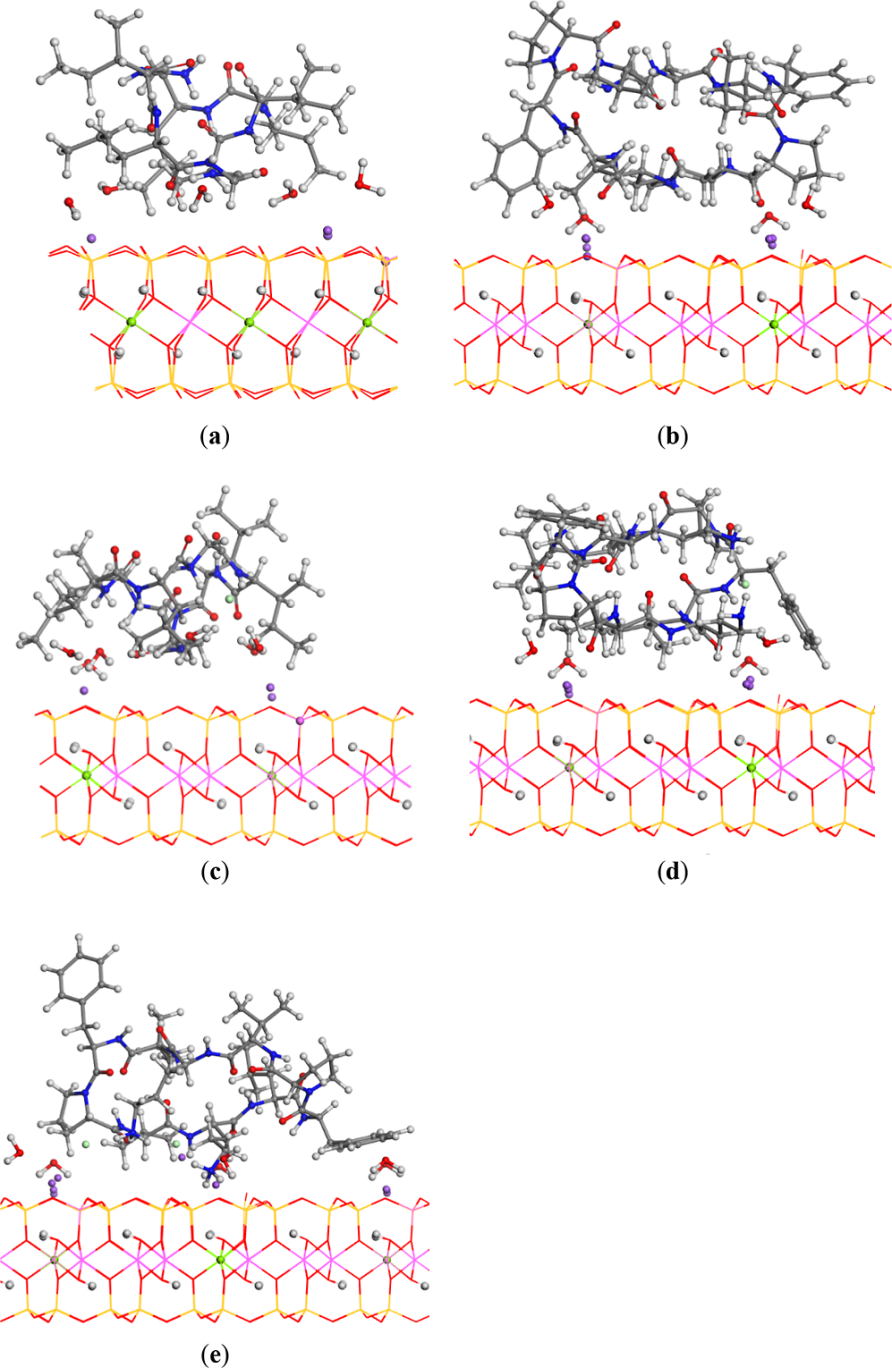
Optimized structures of cyclic peptides onto the external surface of MONT (atom colors: H-white; C-gray; O-red; N-blue; Cl-mint green; Na-purple; Al-harlequin green): (a) ribifolin; (b) gramicidin S; (c) ribifolin monohydrochloride; (d) gramicidin S monohydrochloride; and (e) gramicidin S dichloride.

#### 3.2.4 Adsorption and Desorption Energies

The adsorption energy (*E*_ads_) of the neutral peptides was calculated as the difference between the sum of the energies of the individually optimized isolated cyclic peptides (*E*_pep_) and the isolated clay mineral structure (*E*_MONT_), and the total energy of the fully optimized MONT–peptide complex (*E*_MONT‑pep_) (eq 2)­

2 $$E_{ads}=E_{MONT‐pep}-\left(E_{MONT}+E_{pep}\right)$$

A cation-exchange mechanism contributes to the adsorption of peptides in their protonated states. Under acidic conditions, peptides can be protonated at one of their amine groups, forming ammonium cations. These protonated species can subsequently be adsorbed into the MONT interlayer space via cation exchange, replacing the interlayer sodium cations with peptide cations.

The adsorption energy for monochloride species was calculated by summing the energy of the optimized MONT–peptide complex after removing the Na^+^ ion (*E*_MONT‑pepH+_) and the energy of the NaCl ion pair optimized in a periodic water box containing 400 water molecules (*E*_NaCl‑w_) and then subtracting the total energy of the solvated protonated peptide hydrochloride (*E*_pepHCl‑w_) and that of the isolated MONT structure (eq 3). Considering the relationship: *E*_MONT_ + *E*_pepHCl‑w_ = *E*_MONT‑pepH+_ + *E*_NaCl‑w_

3 $$E_{ads}=\left(E_{MONT‐pepH+}+E_{NaCl‐w}\right)-\left(E_{MONT}-E_{pepHCl‐w}\right)$$

For the doubly protonated systems, the adsorption energy was calculated by using an analogous approach. In this case, two Na^+^ ions were removed from the MONT structure prior to peptide insertion (*E*_MONT‑pepH_2_+_); the aqueous media also contained two Na^+^ and two Cl^–^ ions (*E*_Na_2_Cl_2_‑w_) to maintain the overall charge neutrality and mass stoichiometry; the peptide was solvated with two chloride counterions in the water box (*E*_pepH_2_Cl_2_‑w_) reacting the MONT with the pristine Na^+^ interlayer cations (eq 4)­

4 $$E_{ads}=\left(E_{MONT‐pepH_{2}+}+E_{{Na}_{2}{Cl}_{2}‐w}\right)-\left(E_{MONT}-E_{pepH_{2}{Cl}_{2}‐w}\right)$$

The calculated adsorption energies indicate that the intercalation into the confined interlayer space is less favorable than the adsorption on external MONT surfaces under the studied conditions except for ribifolin via cation exchange. Gramicidin exhibits higher energetic penalties for interlayer insertion than ribifolin (Table 3). Adsorption on external MONT surfaces is exothermic, as described in eq 2, reflecting a favorable energetic tendency and a relative preference for surface adsorption over interlayer regions for both peptides.

**Table 3:** Adsorption and Desorption Energies (in kcal/mol) of Cyclic Peptides on the MONT Surfaces

peptides		interlayer space	external surface	desorption
ribifolin	neutral	20.07	–112.70	–142.75
	monochloride^a^	–5.03	–60.62	–162.49
gramicidin S	neutral	250.41	–47.11	–370.10
	monochloride^a^	259.17	–62.05	–417.31
	dichloride^a^	125.63	–116.23	–345.12

a Cation-exchange intercalation.

The desorption energy (*E*_des_) in the interlayer region provides insight into the retention preference of the peptides within the medium. This energy was calculated using different approaches depending on the system considered: neutral peptides (eq 5), monohydrochloride species (eq 6), and dihydrochloride species (eq 7)­

5 $$E_{des}=\left(E_{w‐pep}+E_{MONT}\right)-\left(E_{w}-E_{MONT‐pep}\right)$$

6 $$E_{des}=\left(E_{w‐pep}+E_{MONT}\right)-\left(E_{NaCl‐w}-E_{MONT‐pep}\right)$$

7 $$E_{des}=\left(E_{w‐pep}+E_{MONT}\right)-\left(E_{2NaCl‐w}-E_{MONT‐pep}\right)$$

Desorption from MONT surfaces is exothermic for all modeled systems, indicating a favorable energetic tendency for the transfer of peptide to the surrounding aqueous phase under the studied conditions. Within the adopted computational framework, both cyclic peptides, therefore, exhibit a propensity to move from the clay mineral surface into the solvent. The present results reflect neutral and acidic pH conditions, and variations in experimental parameters, such as concentration, temperature, and solvent composition, may influence the desorption behavior. In this context, the use of nonaqueous solvents with lower polarity than water has been reported as a strategy to promote peptide intercalation within the MONT interlayer.^31^

Overall, the adsorption, solvation, and desorption energies reported here represent only the initial and final steps of these processes, and these results should be interpreted as effective descriptors rather than as a rigorous thermodynamic decomposition of adsorption or intercalation free energies. Peptide incorporation into layered clays also involves additional effects not captured here, including interlayer reorganization, water-related entropic contributions, ionic redistribution, and kinetic barriers.^79–82^ Nevertheless, this approach enables a consistent comparison of relative affinities and trends across peptides, protonation states, and adsorption environments. Despite the absence of direct experimental validation, the results agree with trends reported in the literature, supporting their use as qualitative descriptors.^10,43^

## 4 Conclusions

This study provides theoretical insight into the behavior of cyclic peptides at MONT surfaces, interlayer space, and external surfaces, under neutral and acidic conditions, including the simulation of cation-exchange mechanisms. The validated methodology showed that the INTERFACE force field yielded results consistent with experimental observations, supporting its suitability for modeling cyclic peptides. The analyses indicate that adsorption onto the external surface of MONT is energetically favorable, whereas intercalation into the interlayer space is thermodynamically unfavorable under fully hydrated conditions. Nevertheless, this process may be enhanced experimentally by controlling the temperature, pH, and reaction time. The predicted increase in the *d*(001) spacing upon intercalation provides useful guidance for future experimental studies. Across all environments, the cyclic topology of ribifolin and gramicidin S is preserved, indicating an intrinsic structural stability consistent with their known biological activity. The energetically favorable release of these peptides from MONT into aqueous media highlights this nanomaterial as a promising platform for peptide delivery and for enhancing the biological performance of cyclic peptide-based therapeutics. The present computational framework offers a solid basis for future theoretical and experimental investigations.

## Supplementary Material



## Notes

MSD profiles for ribifolin intercalated into montmorillonite, including contributions from C, N, carbonyl O, water O, and Na atoms obtained from molecular dynamics simulations; relative concentration profiles along the (001) direction of the montmorillonite interlayer space for ribifolin and gramicidin S derived from molecular dynamics simulations; descriptions of molecular dynamics movies (PDF). Molecular dynamics simulation movies showing ribifolin and gramicidin S intercalated within the montmorillonite interlayer space (AVI) (ZIP)

## Notes

Lucas H. N. Sousa—Instituto de Química, Universidade Federal do Rio Grande do Norte, Natal 59,078-970, Brazil. Claro Ignacio Sainz-Díaz—Instituto Andaluz de Ciencias de la Tierra, Claro Ignacio Sainz-Díaz—Instituto Andaluz de Ciencias de la Tierra, Consejo Superior de Investigaciones Científicas, IACT-CSIC, Av. De las Palmeras, 4, 18100-Armilla, Granada, Spain. César Viseras—Departamento de Tecnología Farmacéutica. Facultad de Farmacia, Universidad de Granada, Granada, Spain

## Abbreviations

MONT: montmorillonite
SPPS: solid-phase peptide synthesis
MS: mass spectrometry
NMR: nuclear magnetic resonance
XRD: X-ray diffraction
CCDC: Cambridge Crystallographic Data Centre
CIF: crystallographic information files
FF: force field
QEq: charge equilibration
