# Supplementary material for: Interactions of Cyclic Peptides Ribifolin and Gramicidin S with Montmorillonite Surface by Molecular Modeling
Source: ACS Omega. 2026 Mar 19;11(12):19523–33. doi: 10.1021/acsomega.5c13251 (PMC13044619; doi:10.1021/acsomega.5c13251)
Supplement: Supplementary file 1 [file ao5c13251_si_001.zip › Supporting Information/Supporting Information.docx]

Interactions of cyclic peptides Ribifolin and Gramicidin S with montmorillonite surface by molecular modelling

Lucas H. N. Sousa^1^, Claro Ignacio Sainz-Díaz^2^, César Viseras^3^, and Renata M. Araújo^1^*

**SUPPORTING INFORMATION**


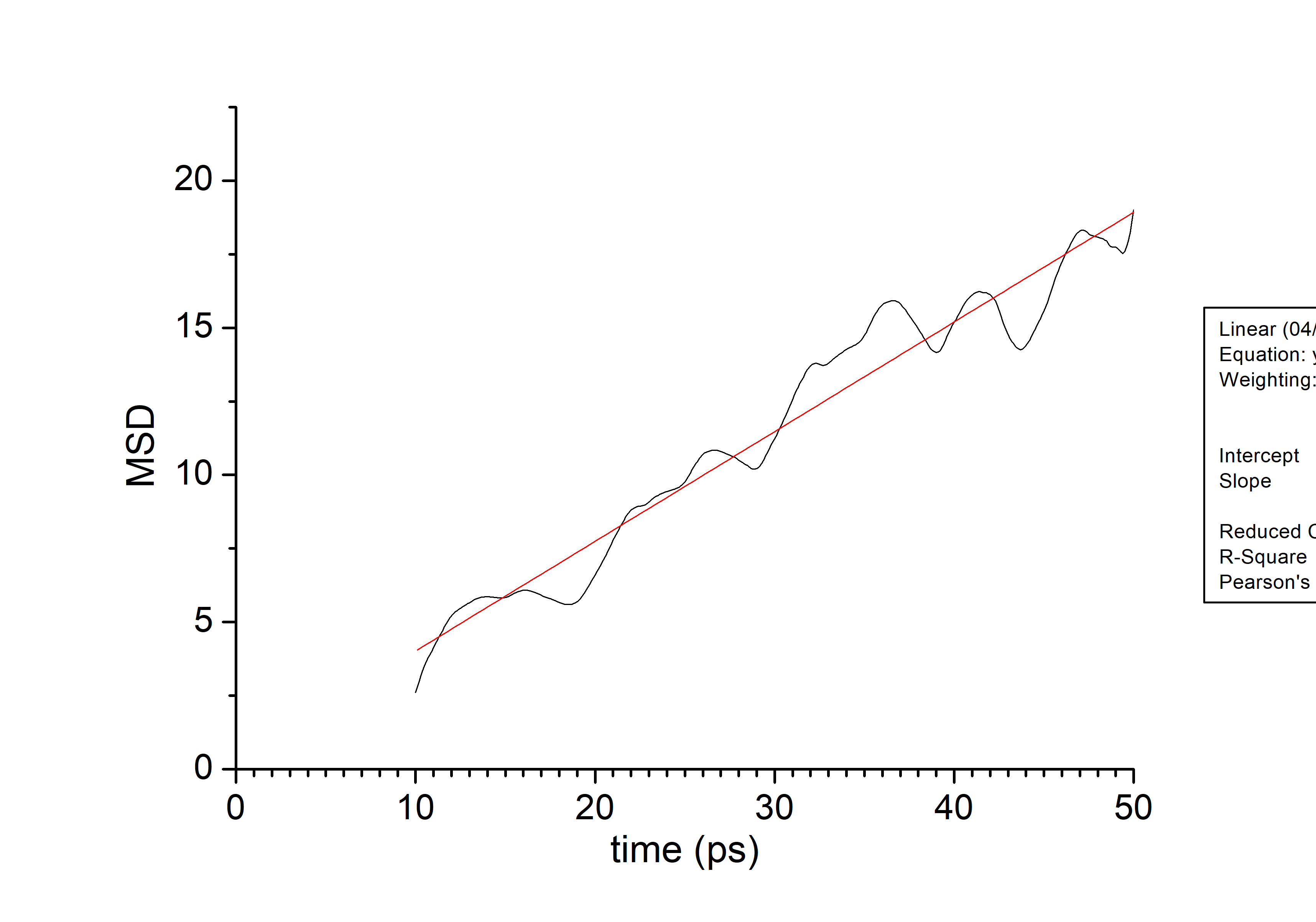
**(a)**
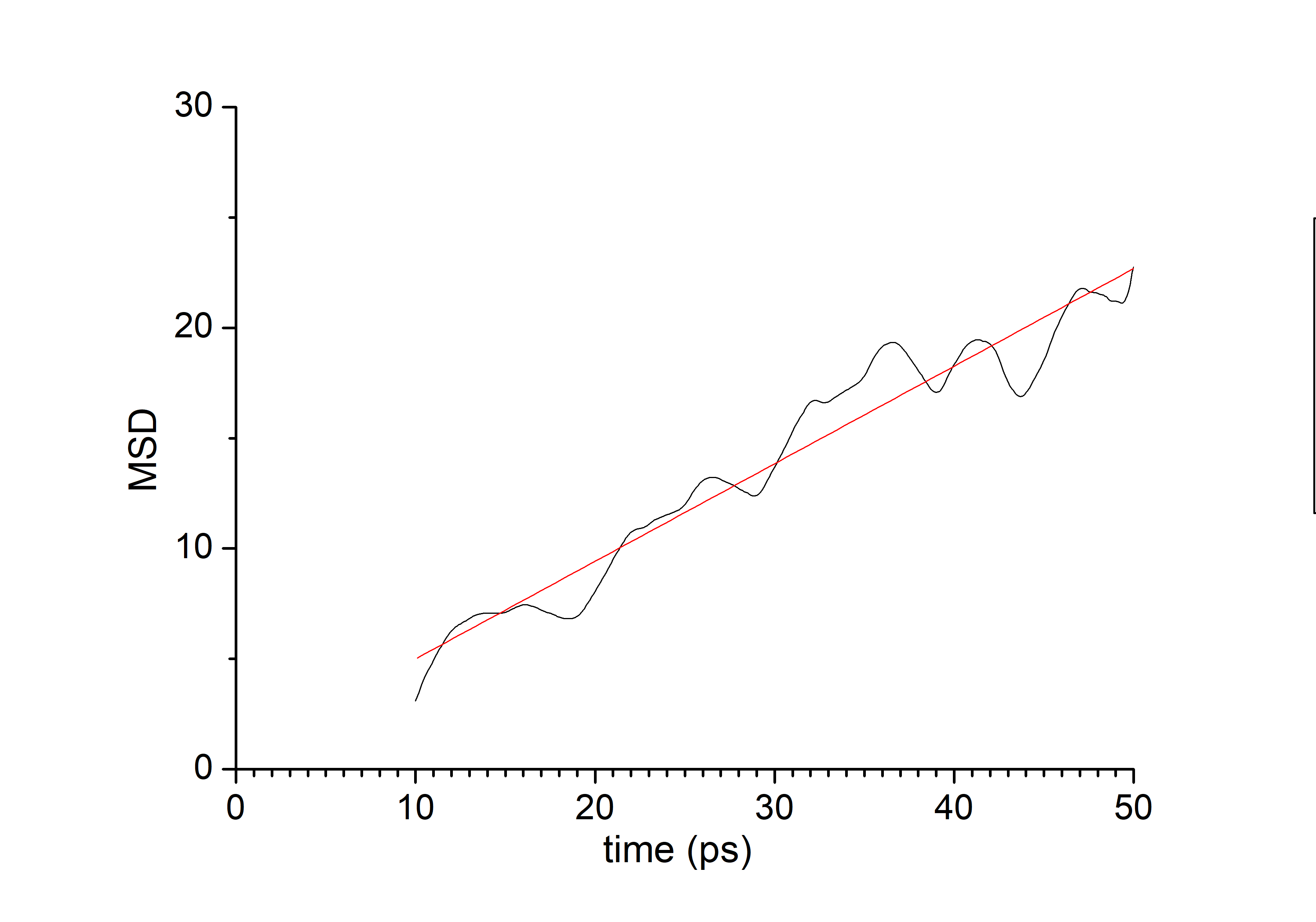
 **(b)**
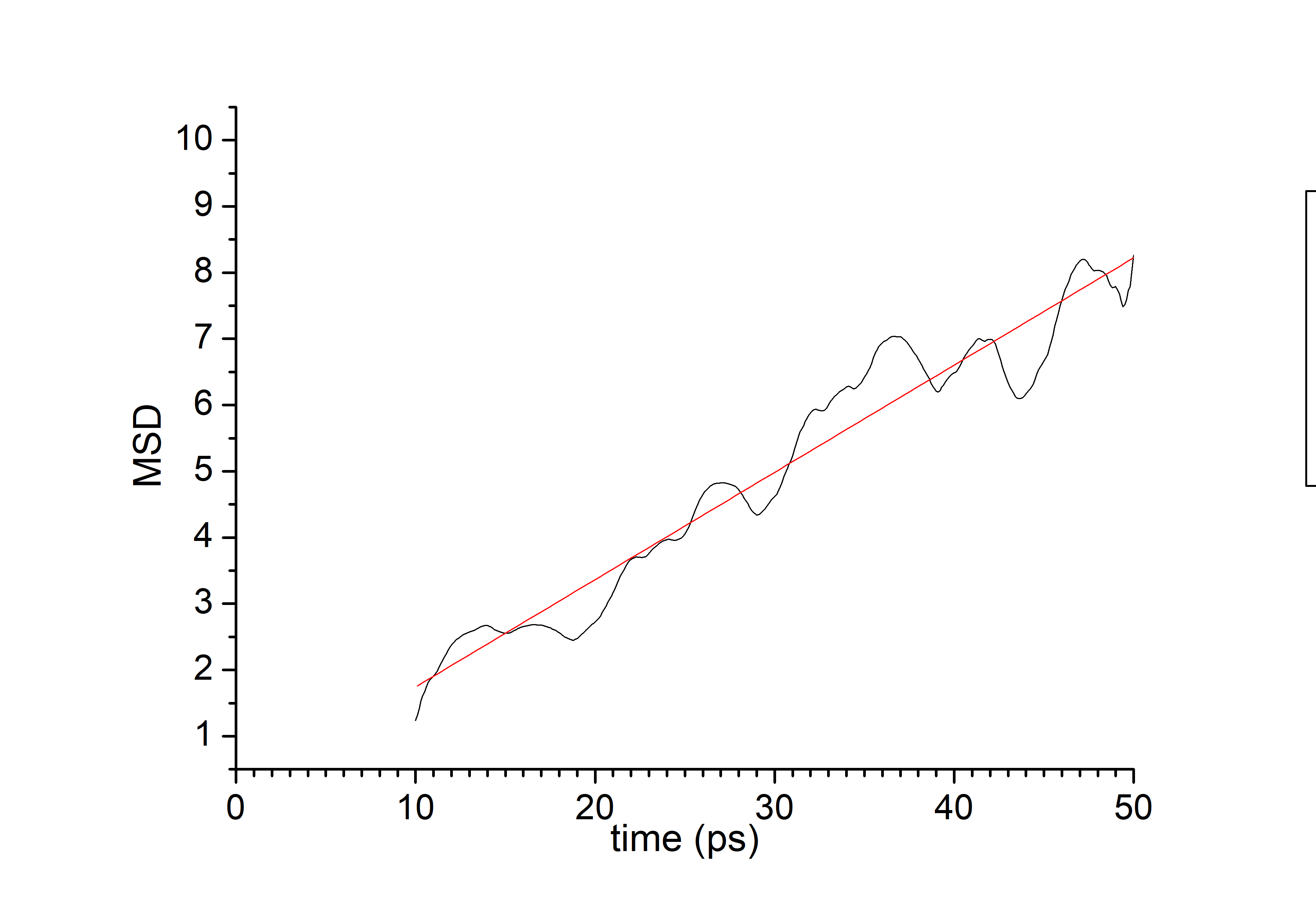
 **(c)**
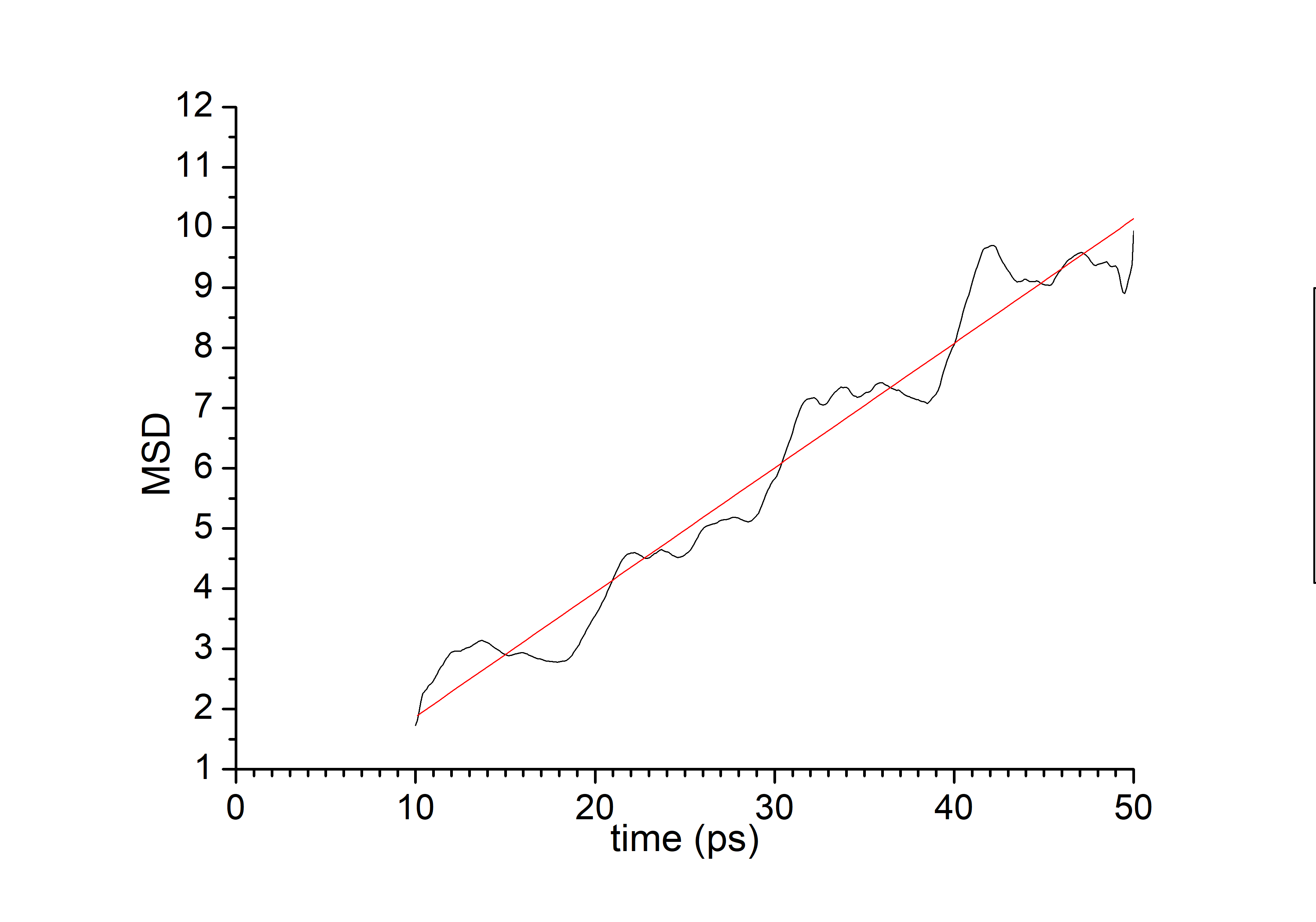
 **(d)**
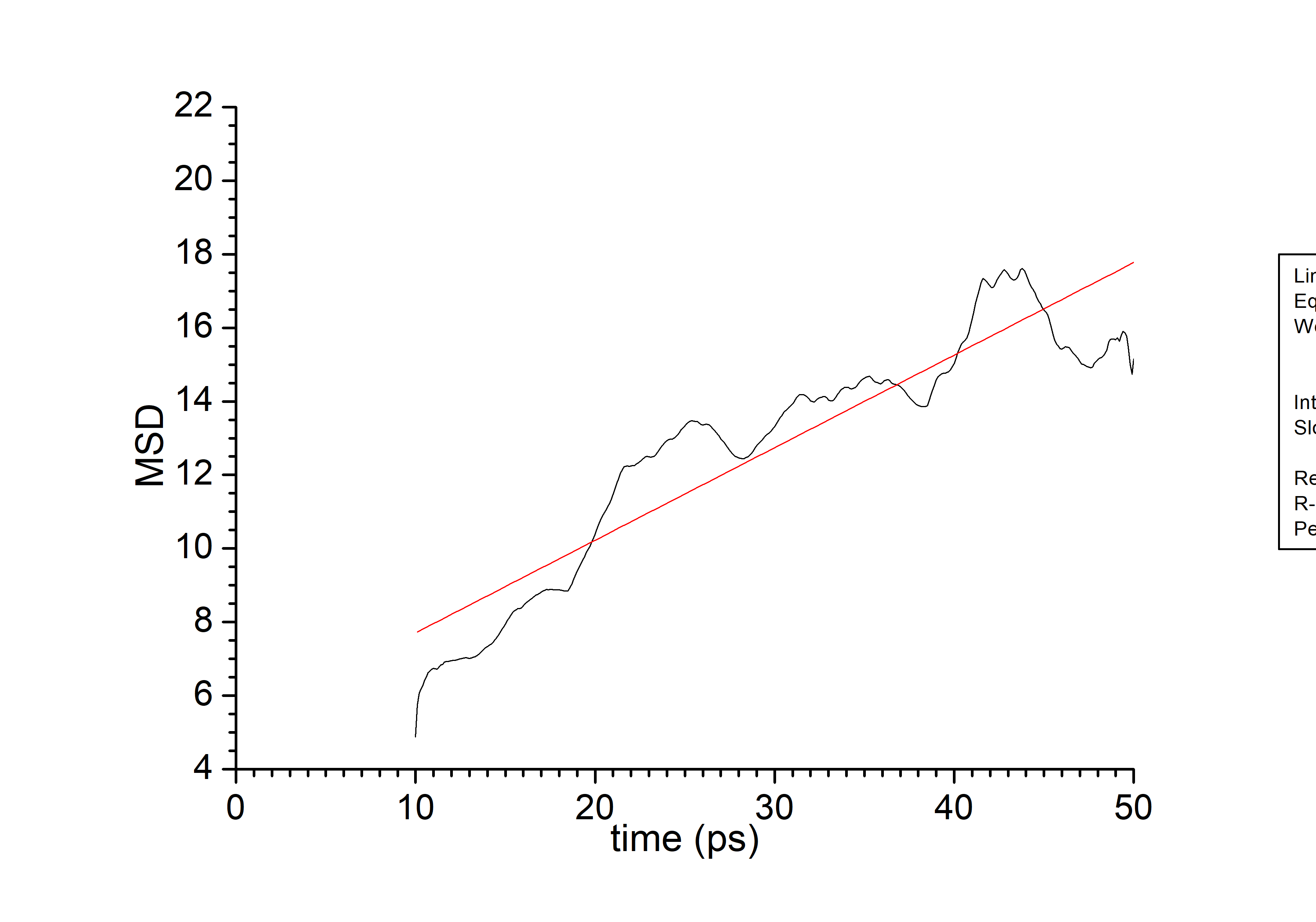
**(e)**
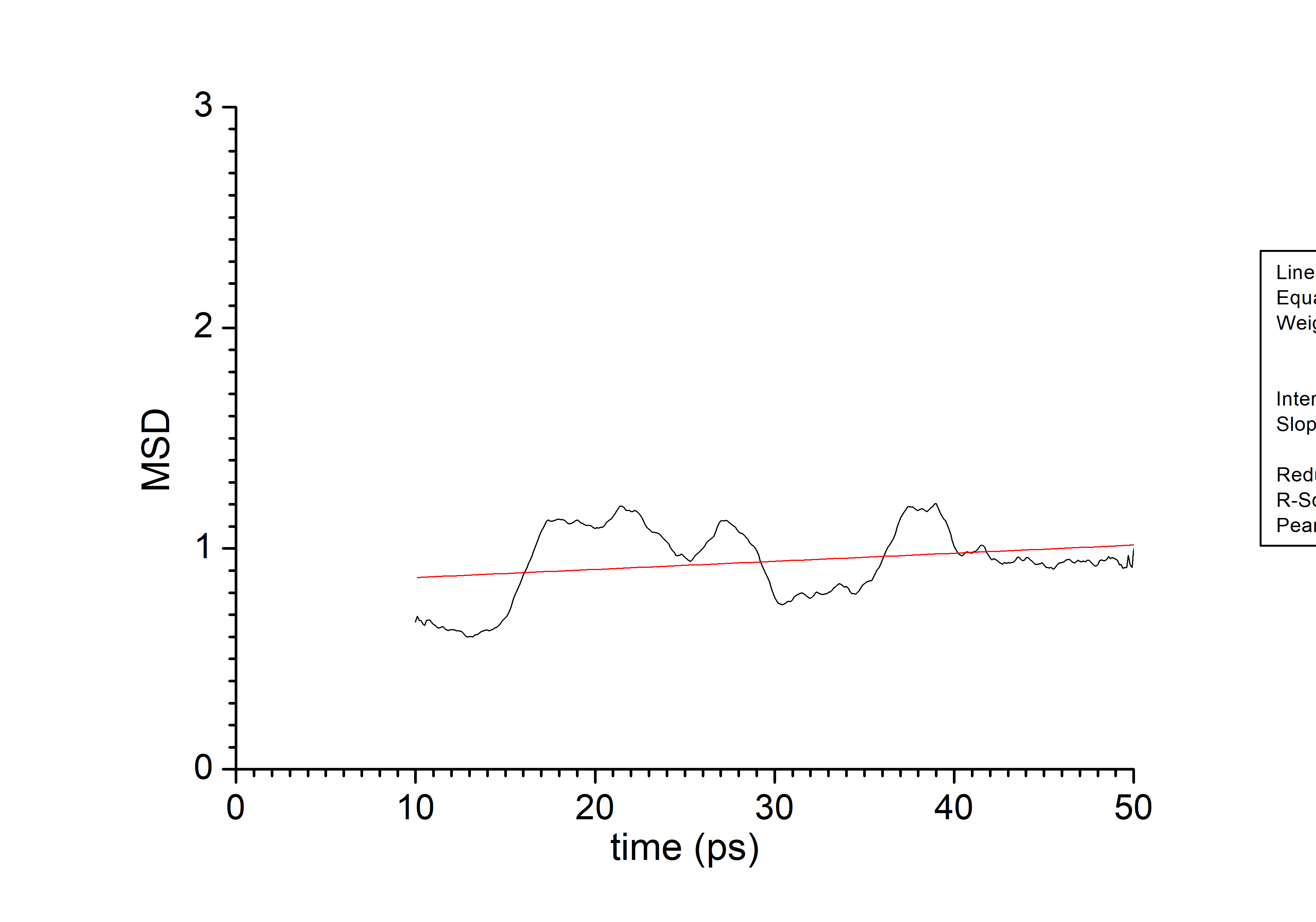
**(f)**

**Figure S1.** Mean Square Displacement profiles of ribifolin (C+N+O atoms) (a), C atoms (b), N atoms (c), carbonyl O atoms (d), water O atoms (e), and Na atoms (f) from molecular dynamics of ribifolin intercalated into MONT. The fitted slope lines are in red color.


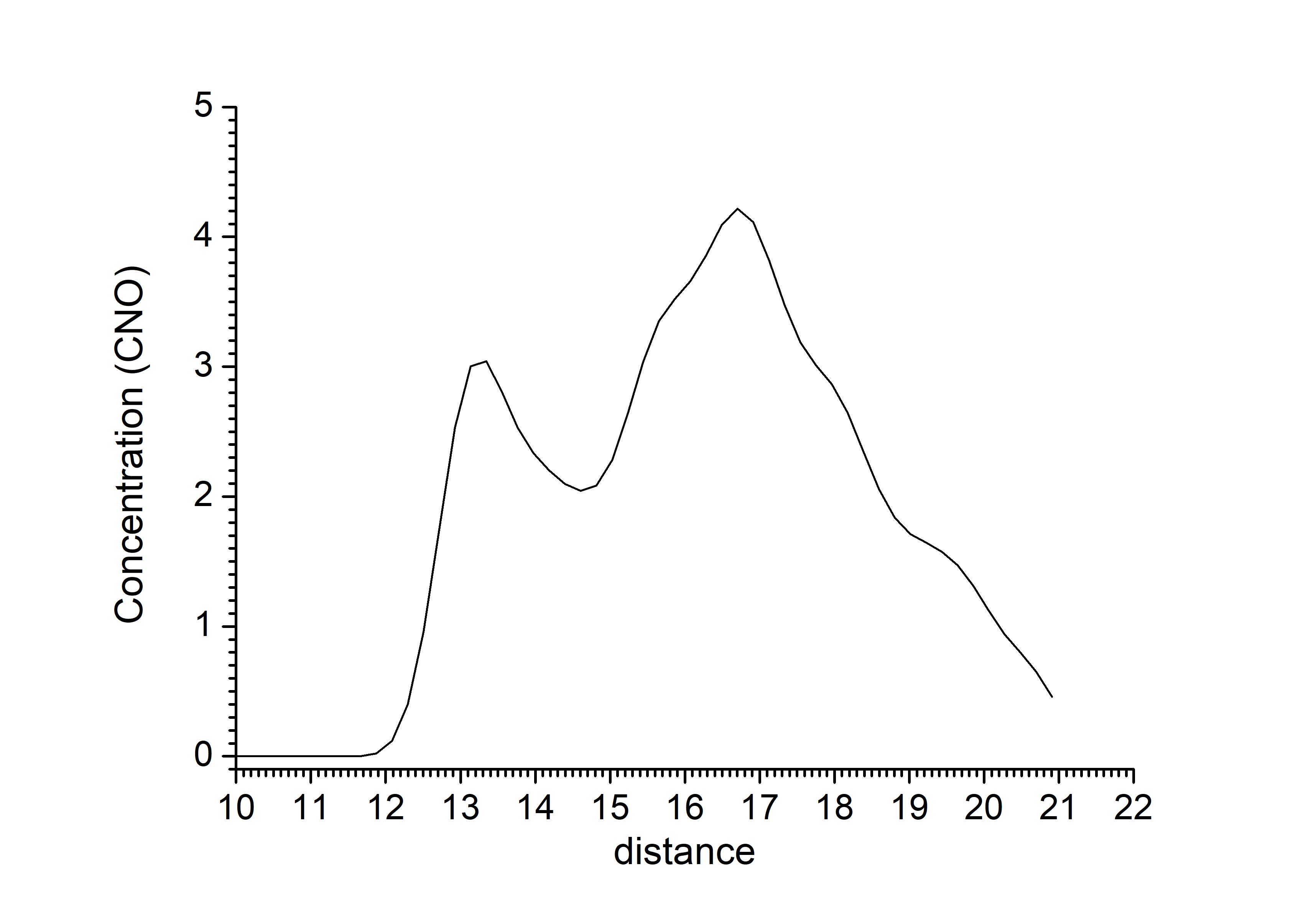

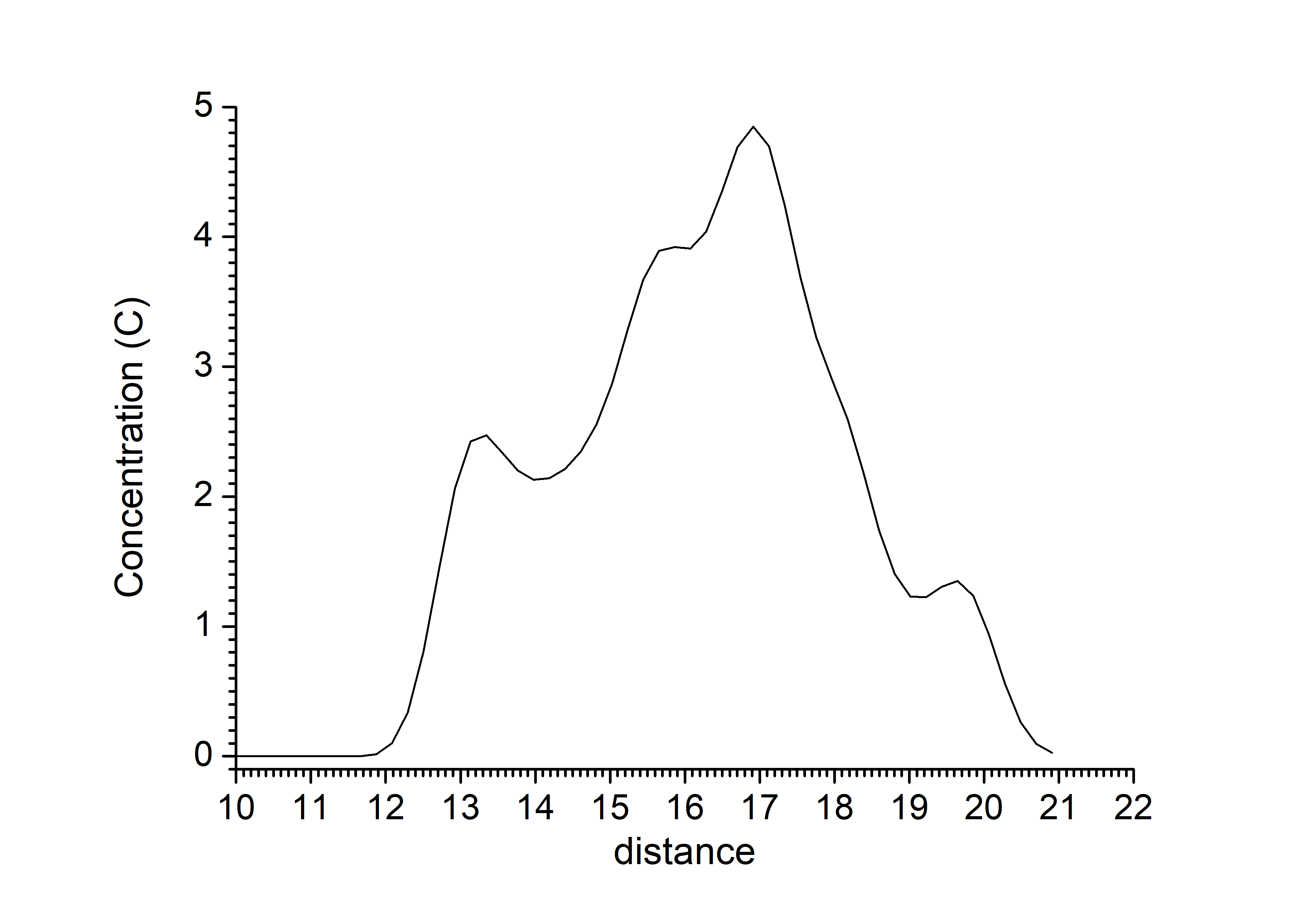


1. **(b)**


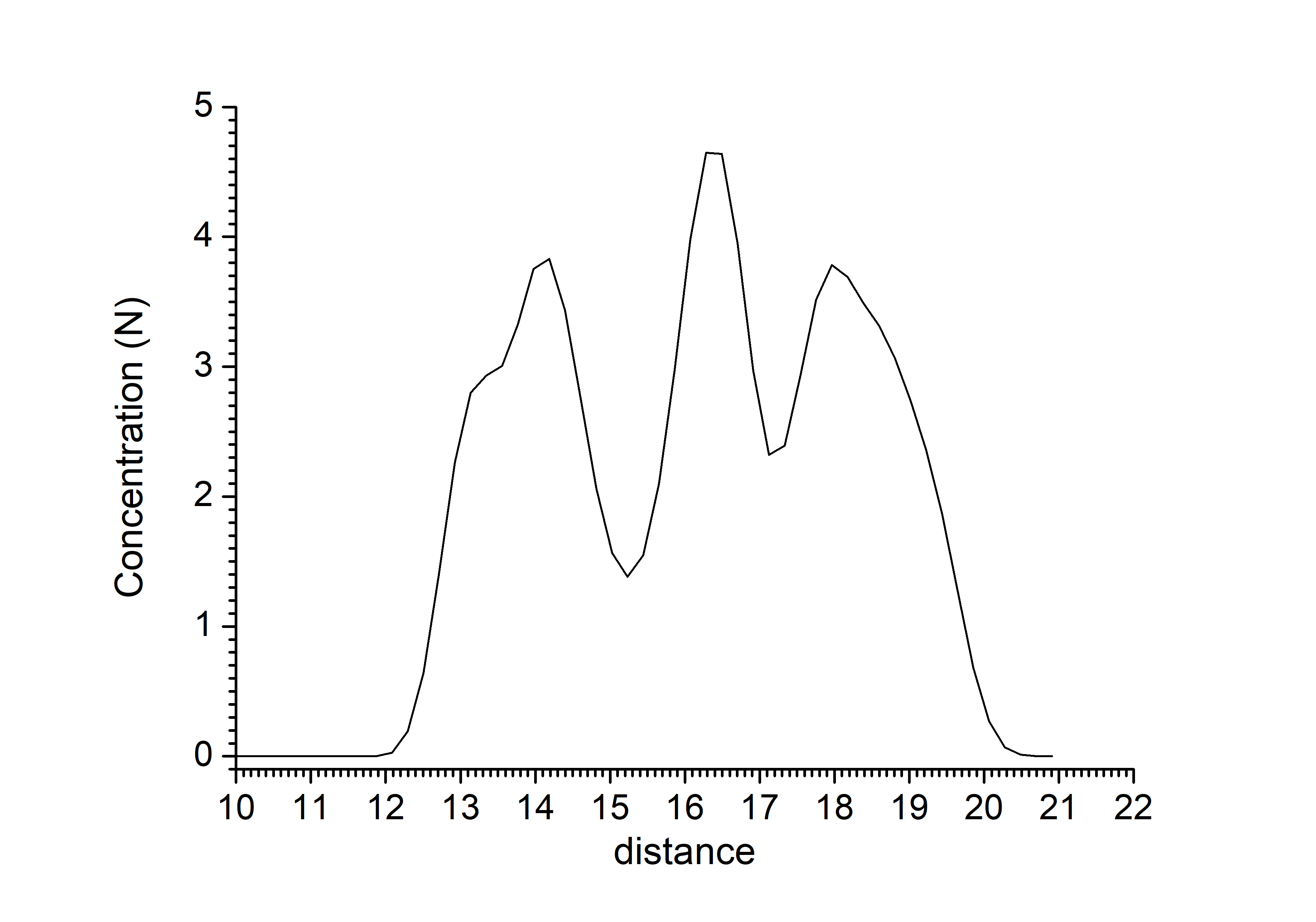
c
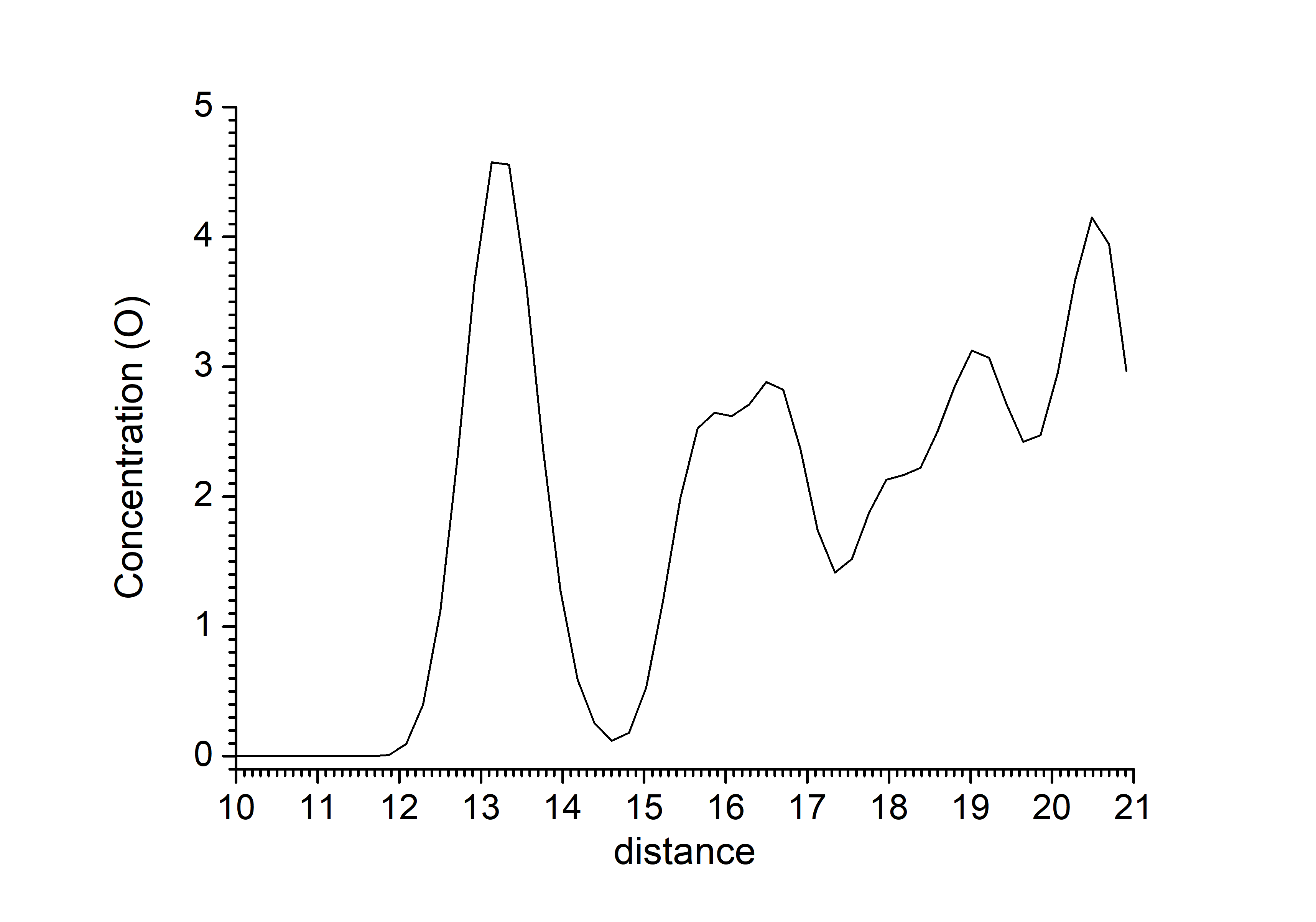


**(c)** **(d)**

**Figure S2**. Relative concentration profiles of ribifolin (C+N+O atoms) (a), C (b), N (c), and peptide O atoms (d) along the (001) direction of the MONT interlayer space for ribifolin intercalated in MONT from MD simulations (distances in Å; the interlayer space is in the 9 to 22 Å distance range).


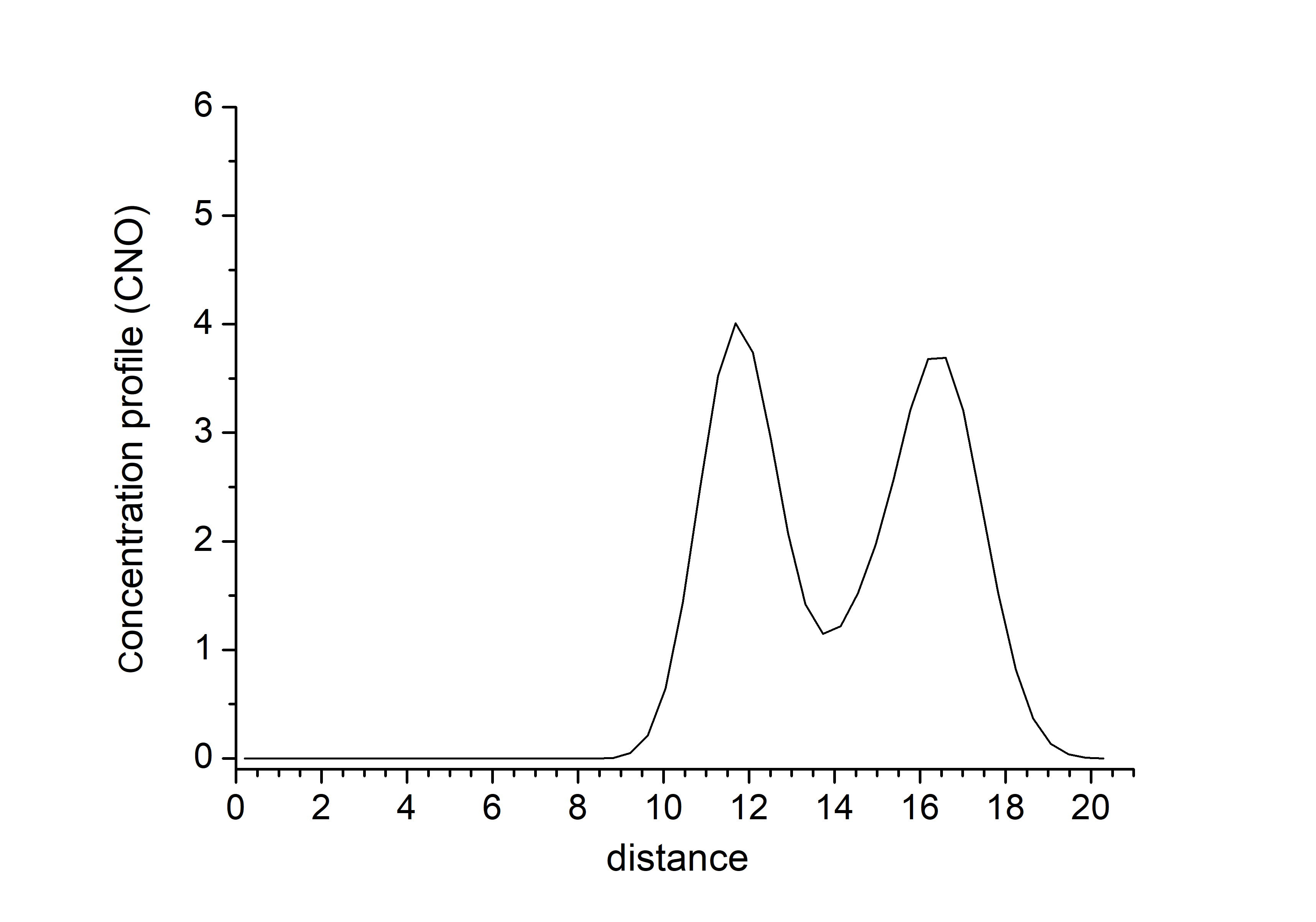


**Figure S3**. Relative concentration profile of gramicidin S (C+N+O atoms) intercalated into MONT along the 001 direction of the crystal structure from MD simulations (distances in Å; the interlayer space is in the 8 to 22 Å distance range).

**Movie M1**.- Molecular dynamics of Ribifolin intercalated into MONT interlayer.

**Movie M2**.- Molecular dynamics of Gramicidin S intercalated into MONT interlayer.
